# Supplementary material for: Causal Relationship between Aging and Anorexia Nervosa: A White-Matter-Microstructure-Mediated Mendelian Randomization Analysis
Source: Biomedicines. 2024 Aug 16;12(8):1874. doi: 10.3390/biomedicines12081874 (PMC11351342; doi:10.3390/biomedicines12081874)
Supplement: Supplementary file 1 [file biomedicines-12-01874-s001.zip › Supplementary Figure/Figure S4/Singe SNP plot of MR analysis for TL to ubm-b-1628.pdf]

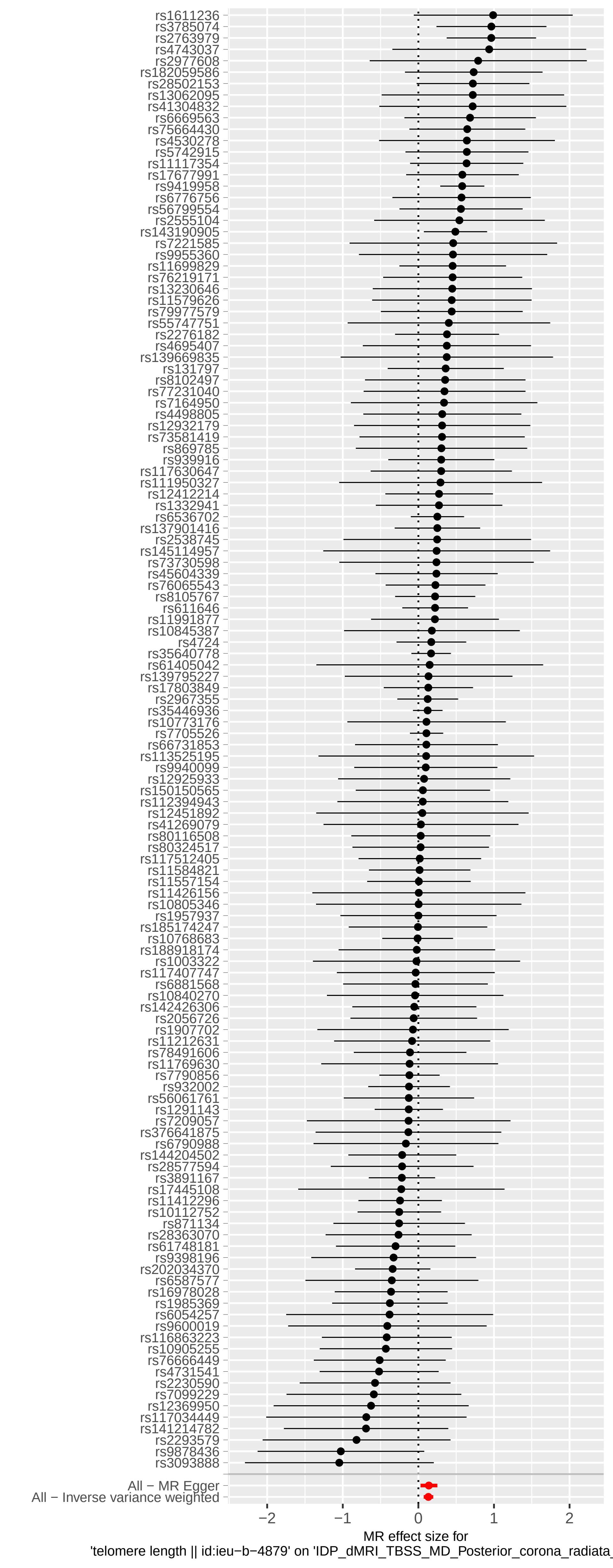
rs1611236
rs3785074
rs2763979
rs4743037
rs2977608
rs182059586
rs28502153
rs13062095
rs41304832
rs6669563
rs75664430
rs4530278
rs5742915
rs11117354
rs17677991
rs9419958
rs6776756
rs56799554
rs2555104
rs143190905
rs7221585
rs9955360
rs11699829
rs76219171
rs13230646
rs11579626
rs79977579
rs55747751
rs2276182
rs4695407
rs139669835
rs131797
rs8102497
rs77231040
rs7164950
rs4498805
rs12932179
rs73581419
rs869785
rs939916
rs117630647
rs111950327
rs12412214
rs1332941
rs6536702
rs137901416
rs2538745
rs145114957
rs73730598
rs45604339
rs76065543
rs8105767
rs611646
rs11991877
rs10845387
rs4724
rs35640778
rs61405042
rs139795227
rs17803849
rs2967355
rs35446936
rs10773176
rs7705526
rs66731853
rs113525195
rs9940099
rs12925933
rs150150565
rs112394943
rs12451892
rs41269079
rs80116508
rs80324517
rs117512405
rs11584821
rs11557154
rs11426156
rs10805346
rs1957937
rs185174247
rs10768683
rs188918174
rs1003322
rs117407747
rs6881568
rs10840270
rs142426306
rs2056726
rs1907702
rs11212631
rs78491606
rs11769630
rs7790856
rs932002
rs56061761
rs1291143
rs7209057
rs376641875
rs6790988
rs144204502
rs28577594
rs3891167
rs17445108
rs11412296
rs10112752
rs871134
rs28363070
rs61748181
rs9398196
rs202034370
rs6587577
rs16978028
rs1985369
rs6054257
rs9600019
rs116863223
rs10905255
rs76666449
rs4731541
rs2230590
rs7099229
rs12369950
rs117034449
rs141214782
rs2293579
rs9878436
rs3093888
All − MR Egger
All − Inverse variance weighted
−2
−1
0
1
2
MR effect size for
'telomere length || id:ieu−b−4879' on 'IDP_dMRI_TBSS_MD_Posterior_corona_radiata_
